# The impact of the COVID-19 pandemic and associated suppression measures on the burden of tuberculosis in India

**DOI:** 10.1186/s12879-022-07078-y

**Published:** 2022-01-27

**Authors:** Matthew Arentz, Jianing Ma, Peng Zheng, Theo Vos, Christopher J. L. Murray, Hmwe H. Kyu

**Affiliations:** 1 Department of Global Health, University of Washington, Seattle, USA; 2 Institute for Health Metrics and Evaluation, Seattle, USA; 3 Department of Health Metrics Sciences, University of Washington, Seattle, USA

## Abstract

**Background:**

Tuberculosis (TB) is a major cause of death globally. India carries the highest share of the global TB burden. The COVID-19 pandemic has severely impacted diagnosis of TB in India, yet there is limited data on how TB case reporting has changed since the pandemic began and which factors determine differences in case notification.

**Methods:**

We utilized publicly available data on TB case reporting through the Indian Central TB Division from January 2017 through April of 2021 (prior to the first COVID-19 related lockdown). Using a Poisson model, we estimated seasonal and yearly patterns in TB case notification in India from January 2017 through February 2020 and extended this estimate as the counterfactual expected TB cases notified from March 2020 through April 2021. We characterized the differences in case notification observed and those expected in the absence of the pandemic by State and Territory. We then performed a linear regression to examine the relationship between the logit ratio of reported TB to counterfactual cases and mask use, mobility, daily hospitalizations/100,000 population, and public/total TB case reporting.

**Results:**

We found 1,320,203 expected cases of TB (95% uncertainty interval (UI) 1,309,612 to 1,330,693) were not reported during the period from March 2020 through April 2021. This represents a 63.3% difference (95% UI 62.8 to 63.8) in reporting. We found that mobility data and average hospital admissions per month per population were correlated with differences in TB case notification, compared to the counterfactual in the absence of the pandemic (p > 0.001).

**Conclusion:**

There was a large difference between reported TB cases in India and those expected in the absence of the pandemic. This information can help inform the Indian TB program as they consider interventions to accelerate case finding and notification once the pandemic related TB service disruptions improve. Mobility data and hospital admissions are surrogate measures that correlate with a greater difference in reported/expected TB cases and may correlate with a disruption in TB diagnostic services. However, further research is needed to clarify this association and identify other key contributors to gaps in TB case notifications in India.

**Supplementary Information:**

The online version contains supplementary material available at 10.1186/s12879-022-07078-y.

## Background

In 2019, an estimated 10 million individuals developed active TB, and it was the most common cause of death from a single infectious agent globally [1, 2]. India is the second most populous country in the world, and carries the highest share of the TB burden, accounting for 26% of the estimated new cases globally in 2019 [2, 3]. Prior to the pandemic, efforts in India to decrease the gap in TB case finding had demonstrated promise, with an increase in TB case finding of 60% from 2013 to 2019 [4].

The COVID-19 pandemic has upended TB control efforts, and has impacted resource allocation for TB diagnosis, treatment, and prevention [5, 6]. Modeling estimates from early in the pandemic demonstrated that COVID-19 control measures may have a large impact on TB transmission, incidence, and death in the coming years, with a marked increase in TB related deaths and a profound expected setback in TB control [7, 8]. Early available data on case notification from India was troubling, and demonstrated a 70% decrease in case reporting between weeks 10 and 15 of 2020 in comparison to 2019 [6].

Because the COVID-19 pandemic has rapidly evolved, relatively few estimates of how COVID-19 suppression measures have impacted TB testing and diagnosis exist. Furthermore, although surveys have indicated that TB services have been interrupted through reallocation of staff and resources, no variables that are longitudinally available have been identified which correlate with these service interruptions and might help better understand when and where gaps in TB care exist [5]. In India, the Central TB Division of the Government of India has provided publicly available data on TB case notification, but no comprehensive estimate of TB disease in the setting of the pandemic exists and no measures exist which characterize how TB diagnosis and notification of cases are impacted by lockdown measures [9].

There are few studies that characterize TB seasonal patterns in India, and those available reported observations prior to 2012 [10–12]. In this study, we leveraged monthly TB case reporting data from Indian states and territories to characterize the seasonal pattern in TB case reporting over time and estimated the counterfactual TB case notification for the period from March 2020 to April 2021 if there had been no COVID-19 pandemic. We then examine the contribution of various COVID-19 control measures in explaining the difference between the expected TB cases reported in Indian states, and the actual number reported from March of 2020 to April of 2021.

## Methods

### Input data

Nikshay is the online publicly available TB case reporting and surveillance system through the Central TB Division of the Ministry of Health and Family Welfare for the Indian Government [9]. This system allows access to public and private sector TB case reporting over time in India. TB case reporting data were abstracted from the Nikshay by month and location (state/territory) from January of 2017 through April of 2021 and all reported TB cases were included in our analysis. Nikshay data for specific states were aggregated to align with Institute for Health Metrics and Evaluation (IHME) India data for further analysis. Specifically, data from Andaman & Nicobar Island, Dadra and Nagar Naveli, Daman, Diu, Chandigarh, Lakshadweep, and Puducherry were aggregated as “Other Union Territories”. Data from Jammu and Kasmir, and data from Ladakh were aggregated into the territories “Jammu, Kashmir, and Ladakh”.

### Independent and dependent variables

Our dependent variable was the ratio of reported TB cases from the Nikshay to the counterfactual number of TB cases expected in the absence of the pandemic as described below. This variable was calculated by state and month from March 2020 through April of 2021. To better understand which independent variables correlated with subnational trends in TB case notification gaps, we utilized the IHME’s estimates of control measures for India from the COVID-19 modeling strategy [13]. Our independent variables were mobility, mask use, average monthly COVID-19 hospital admissions per 100,000 population, and ratio of public to total case notification by state/territory by month from March 2020 to April of 2021. The population estimates were from the Global Burden of Diseases, Injuries, and Risk Factors Study [14]. The mobility covariate is measured as the relative change from pre-pandemic baseline using data from Google and Facebook and is further described in Additional file 2: Appendix S2.

A positive value indicates mobility would exceed pre-pandemic baseline and a negative value indicates that mobility has decreased below pre-pandemic baseline measures [15]. Mobility estimates were averaged over each month of the analysis. Mask use in India is estimated using facemask use reported from the Facebook global symptom survey (based on a survey from the University of Maryland Social Data Science Center) and is represented as a mean probability of mask use. COVID-19 hospital admissions per day per 100,000 population were averaged by month for each state using the IHME COVID-19 modeling estimates [15]. Data on mask use, mobility, and COVID-19 hospital admissions were present for all states and territories in India other than the Union Territories. Private and public TB case notifications are available through the Nikshay database and public/total case notification ratios were also calculated by state and month [9].

We rationalized that either a decrease in mobility or an increase in mask use would be associated with a decreased ability to access TB diagnosis and care due to interruption in access to health services. However, we also acknowledged that an increase in mask use could also be associated with reduced transmission and incidence of TB and subsequent reporting. We also rationalized that an increase in COVID-19 hospital admissions would increase the strain on health systems, and decrease allocations of services for TB care, thus decreasing case notification. Lastly, it was postulated that private health systems in India would be less likely to diagnose and notify TB cases due to strains in health system capacity from the COVID-19 pandemic. It has been previously noted that private sector providers are less likely notify TB cases than public sector providers due to logistic barriers in screening and notification [16]. We therefore reasoned that the increase strain of COVID-19 cases would more severely impact TB screening and notification among this group. This was expected to result in a decrease in case notification in the private sector. Therefore, our hypothesis was that the ratio of public to total case notification (i.e., a combination of private and public notification) would increase in states with a larger gap in reported to expected case notification.

### Data analysis

TB case reporting data from January 2017 through February 2020 (prior to the population-wide lockdown instituted in March of 2020) were analyzed using a sequential Poisson model to examine the seasonality pattern and the overall time trend of TB case notifications. To obtain the seasonality pattern, data were first grouped by month across years. We then ran a Poisson model using population as an offset and a spline across months with three interior knots (placed in March, June, and September) to estimate the seasonality pattern*.* We avoided adding more knots as this can lead to overfitting and unstable model behavior without changing the seasonality pattern much within the quarter. Next, we ungroup the monthly data, predict out the cases for each month from the first Poisson model and use it as the offset in a second Poisson model to estimate the overall time trend. We use sandwich estimation approach to incorporate both uncertainty from the second stage Poisson model and the uncertainty remaining in the final residual to generate uncertainty intervals and represented these in our results at the 2.5th and 97.5th percentiles [17]. This model was then extrapolated to estimate counterfactual expected case notifications in the absence of the pandemic from March of 2020 to April of 2021 by month and by state/territory. A critical assumption of this model is that historical trends from 2017 to early 2020 would be expected to continue in later 2020 and in 2021 in the absence of the pandemic. The ratio of monthly reported to expected case notification was calculated by state and territory from March of 2020 to April of 2021 (after lockdown measures were instituted). A linear regression was then performed to examine the relationship between the logit ratio of reported TB cases to counterfactual expected cases (our dependent variable) and mask use, mobility, daily hospital admissions/100,000 population, and the public/total TB case reporting ratio (our independent variables). The “vce(cluster)” option in Stata was used to produce cluster-robust standard errors [18]. A Shapley decomposition was performed to quantify the contribution of each independent variable to variance in this regression model [19, 20].

## Results

### Trends in TB case reporting and gaps in reported cases during the COVID-19 pandemic

**Fig. 1:**
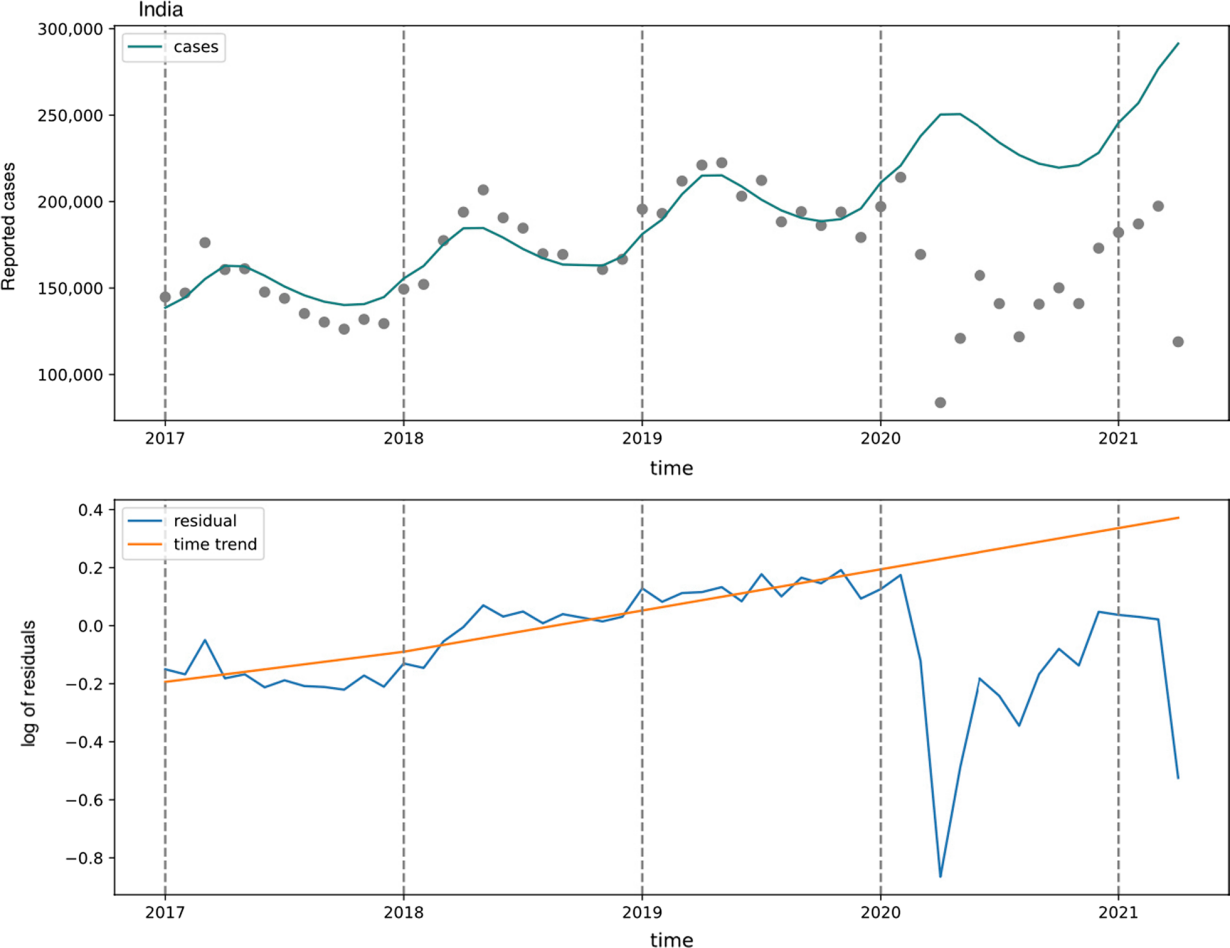
TB case notification and time trend in India, January 2017 to April 2021. Top panel—Case notification in India from January 2017 to April 2021. Grey points represent case notification by month. The teal line represents a model fit to case notification seasonal and year trends from January 2017 to February 2020, prior to pandemic lockdown measures in March of 2020. This trend was then extended from March 2020 to April 2021 as the counterfactual expected cases in the absence of the pandemic (March 2020 to April 2021). Bottom panel—time trend of expected cases (orange line) fit to residuals (blue line) in India, January 2017 to April 2021

Over the period from January 2017 to February 2020 there was a yearly trend towards increased case notification with significant seasonal variation (Fig. 1). Similar trends were observed in State and territory reporting. There were 2,084,522 reported cases of tuberculosis in India from March 2020 to April 2021. Utilizing our counterfactual TB case notifications in the absence of the pandemic, there would have been 3,404,725 expected cases in the absence of the COVID-19 pandemic. This difference represents a 63.3% decrease in case notification (Table 1). All states and territories had a gap in reported to expected case reporting, and the % decrease ranged from 10.9% (in Sikkim) to 114.9% (in the Union Territories) (Table 1 and Additional file 1: Appendix S1). Over 50% of the overall difference in reported to expected cases in the absence of the pandemic was observed in four states (Madhya Pradesh, Maharashtra, Rajasthan, and Uttar Pradesh). Delhi had the highest difference in case notification/100,000 population (a difference of 429 case notifications per 100,000 population), followed by Madhya Pradesh (a difference of 144 case notifications per 100,000 population) and Nagaland (a difference of 133 case notification per 100,000 population). Differences in case notification were greater earlier in 2020. Over the first 4 months of the lockdown in India, from March to June 2020, 34% of the total difference in case notification was observed, which correlated with 450,536 notifications.

**Table 1:** Reported and expected TB case reporting March 2020 to April 2021 by state/territory

Location	Reported cases	Expected cases (95% UI)	Difference, expected − reported cases (95% UI)	Percent difference^a^ (95% UI)
Andhra Pradesh	76,618	122,874 (121,020, 124,767)	46,256 (44,402, 48,149)	60.4 (58.0, 62.8)
Arunachal Pradesh	3006	3606 (3317, 3934)	600 (311, 928)	20.0 (10.3, 30.9)
Assam	40,421	71,299 (69,764, 72,892)	30,878 (29,343, 32,471)	76.4 (72.6, 80.3)
Bihar	121,347	166,639 (164,379, 168,966)	45,292 (43,032, 47,619)	37.3 (35.5, 39.2)
Chhattisgarh	30,964	54,942 (53,699, 56,187)	23,978 (22,735, 25,223)	77.4 (73.4, 81.5)
Delhi	101,797	184,301 (181,631, 187,087)	82,504 (79,834, 85,290)	81.0 (78.4, 83.8)
Goa	1911	2933 (2667, 3238)	1022 (756, 1327)	53.5 (39.5, 69.4)
Gujarat	135,235	196,770 (194,518, 199,171)	61,535 (59,283, 63,936)	45.5 (43.8, 47.3)
Haryana	74,433	111,043 (109,110, 113,039)	36,610 (34,678, 38,606)	49.2 (46.6, 51.9)
Himachal Pradesh	15,441	21,682 (20,929, 22,466)	6241(5488, 7025)	40.4 (35.5, 45.5)
Jammu & Kashmir and Ladakh	10,741	16,029 (15,333, 16,747)	5288 (4592, 6006)	49.2 (42.8, 55.9)
Jharkhand	52,592	78,191 (76,623, 79,748)	25,599 (24,031, 27,156)	48.7 (45.7, 51.6)
Karnataka	75,214	120,555 (118,689, 122,497)	45,341 (43,475, 47,283)	60.3 (57.8, 62.9)
Kerala	23,510	34,182 (33,213, 35,223)	10,672 (9703, 11,713)	45.4 (41.3, 49.8)
Madhya Pradesh	156,062	284,847 (281,554, 288,141)	128,785 (125,492, 132,079)	82.5 (80.4, 84.6)
Maharashtra	180,490	299,242 (296,311, 302,248)	118,752 (115,821, 121,758)	65.8 (64.2, 67.5)
Manipur	1822	3002 (2744, 3278)	1180 (922, 1456)	64.8 (50.6, 79.9)
Meghalaya	4842	7706 (7228, 8252)	2864 (2386, 3410)	59.1 (49.3, 70.4)
Mizoram	2550	3870 (3552, 4229)	1320 (1002, 1679)	51.8 (39.3, 65.8)
Nagaland	3788	6278 (6319, 7275)	2690 (2239, 3195)	65.9 (54.9, 78.3)
Odisha	53,852	70,970 (69,528, 72,387)	17,118 (15,676, 18,535)	31.8 (29.1, 34.4)
Punjab	53,329	83,305 (81,653, 84,903)	29,976 (28,324, 31,574)	56.2 (53.1, 59.2)
Rajasthan	158,059	252,607 (249,781, 255,455)	94,548 (91,722, 97,396)	59.8 (58.0, 61.6)
Sikkim	1421	1601 (1549, 1962)	172 (-22, 391)	10.9 (-1.4, 24.9)
Tamil Nadu	80,719	150,202 (148,115, 152,358)	69,483 (67,396, 71,639)	86.1 (83.5, 88.8)
Telangana	69,603	114,807 (112,731, 116,887)	45,204 (43,128, 47,284)	64.9 (62.0, 67.9)
Tripura	2510	3601 (3280, 3937)	1091 (770, 1427)	43.5 (30.7, 56.8)
Uttar Pradesh	426,622	753,951 (748,643, 759,286)	327,329 (322,021, 332,664)	76.7 (75.5, 78.0)
Uttarakhand	22,945	40,675 (39,472, 41,886)	17,730 (16,527, 18,941)	77.3 (72.0, 82.6)
West Bengal	92,542	145,730 (143,747, 147,788)	53,188 (51,205, 55,245)	57.5 (55.3, 60.0)
Other Union Territories	9694	20,836 (19,995, 21,723)	11,142 (10,301, 12,029)	114.9 (106.3, 124.1)
India (total)^b^	2,084,522	3,404,725 (3,394,134, 3,415,214)	1,320,203 (1,309,612, 1,330,693)	63.3 (62.8, 63.8)

*UI *uncertainty interval

^a^Percent difference calculated as [(expected cases – reported cases)/expected cases] * 100

^b^India (total) data calculated from a national model (not aggregate)

### COVID-19 suppression measures, and their association with gaps in reported/expected TB cases

Over the period from March 2020 to April 2021 we evaluated covariates associated with the ratio of reported to expected TB case notification by state and month (Table 2).

**Table 2:** Evaluation of covariates in the regression model

Covariate	Mean	Min, Max	SD	Coefficient	p value	Shapley % R^2^
Mask use	0.597	0.000, 0.858	0.200	0.015	0.927	2.34%
Mobility	− 33.636	− 87.136, − 1.937	16.015	0.024	< 0.001	71.40%
Hospital admissions, per 100 K population	2.291	0.004, 38.843	4.089	− 0.05	< 0.001	23.15%
Public/total case notification ratio	0.765	0.420, 1.000	0.122	0.482	0.552	3.11%

Overall R^2^ was 39.5%. Coefficient for the regression, p value, and percentage contribution to the R^2^ for mask use, mobility, hospital admissions, and the ratio of public case notification/total case notification are demonstrated in the table. Regression was not performed on the Union Territories due to lack of available data on mobility, mask use, and COVID-19 related hospitalizations from that area

*SD* standard deviation

Decreased mobility was associated with a decrease in the ratio of reported to expected cases (suggesting a decrease in access to TB care), and this association was significant (p < 0.001). However, increased mask use did not show an association with a decrease in TB case notification compared to that expected in the absence of the pandemic (p = 0.927). Average monthly COVID-19 hospital admissions per 100,000 were inversely associated with the ratio of reported to expected TB cases, suggesting that an increase in admissions due to COVID-19 was associated with an increase in a gap between reported to expected TB case reporting (p < 0.001). Lastly, we observed an increase in the public to total case notification ratio was associated with a trend towards a decreased gap in case notification. This observation did not support our hypothesis, although the trend was not significant (p = 0.552). The four covariates evaluated explained 39.5% of the variation in the reported/expected case ratio; 71.4% of variance was attributed to mobility and 23.2% to COVID-19 hospital admissions.

A critical assumption of this analysis was that historical trends in case reporting would continue in the absence of the pandemic. We conducted a sensitivity analysis of COVID-19 suppression measures with the stagnant assumption (i.e. reported TB cases from March 2020 to February 2021 would be the same as reported cases from March 2019 to February 2020). This analysis is shown in Additional file 2: Appendix S2.

## Discussion

In this study, we’ve characterized the difference between TB case notification in India from March of 2020 through April of 2021 and the counterfactual case notification expected in the absence of the pandemic. We have been able to quantify a significant difference between the number of TB case notifications during that time period, and the number that would have been expected in the absence of the pandemic based on historical annual and seasonal trends. This study demonstrates that over 1.3 million additional individuals would have been expected to be identified with TB in India over the same time period in the absence of the pandemic. Prior estimates from India and other countries evaluating the drop in TB case notifications in 2020 in the setting of pandemic compared this number of case notifications reported to historical values, typically TB case notifications from 2019 [6, 21–25]. In countries which would have expected an increase in case notification in 2020 compared to 2019, such as India, such historical comparison could underestimate the severity of the notification gap in the setting of the pandemic. Our findings suggest that COVID-19 control measures may have greater lasting implications on future TB case notification and deaths than initially estimated. Our sensitivity analysis results show that these findings still hold when considering the assumption that TB case notification during the pandemic was the same as for the prior year.

However, it is important to consider other factors unique to the COVID-19 pandemic that may impact TB transmission in important ways. It is known that other factors specific to the pandemic, such as mask wearing, social distancing, and decreased mobility may impact TB transmission, and have been shown to decrease the transmission of other infectious including influenza [26]. Human studies demonstrate that if patients with active TB wear masks, there is a decrease in tuberculin skin test conversion and active TB disease among close contacts, and mask use can result in as much as a 14% reduction in TST conversion [27–32]. As a result, in high burden TB countries where mask use is elevated, there may be a protective effect on transmission. Additionally, genetic epidemiology studies have demonstrated that as much as 80% of TB transmission occurs outside of the home, and these transmission dynamics may be interrupted by increased social distancing and decreased mobility [33, 34]. As a result, mask wearing, and decreased mobility may have a role in decreasing community transmission of TB. Conversely, there may be an increase in home transmission of TB given the increased stay at home orders. It is worth noting that a large percentage of the decrease in TB case notification we observed in India occurred in the first 4 months of the lockdown (34% of the total). Although factors such as decreased mobility and mask use may have decreased the cascade of TB transmission to TB infection to active TB disease in certain settings, it is unlikely that these time dependent factors would explain all of the large differences in case notification we characterized in India during the first 4 months of the pandemic. Additionally, it is not well known how individual behavior (such as mobility and government suppression measures) might be associated with transmission dynamics over time. Others have determined that the correlation between mobility data and transmissibility of COVID-19 weakened after the relaxation of stringent control measures, which may have a similar impact on TB transmission [35]. Is clear is that updated TB surveillance data is sorely needed in high burden TB countries as the pandemic continues to better understand how these factors are influencing transmission dynamics. Although it will take time to become available, cause of death data will be a key tool to help characterize how much of the drop in case notifications is due to a decrease in case identification, and how much is due to a decrease in transmission.

Given the ongoing COVID-19 crisis in India and Southeast Asia, and the possibility for another wave of COVID-19 cases in many parts of Africa, it is important that surrogate measures be developed which might allow for better estimates of the impact of COVID-19 control measures on TB case notification. In this study, we found that mobility data and average hospital admissions per month per population were correlated with differences in TB case notification, compared to the counterfactual in the absence of the pandemic. As a result, these factors may be reasonable covariates for estimating a decrease in access to TB diagnosis and treatment and/or a reduction in incident disease, at least in India. However, other factors not included in our model may contribute to the variance seen, as the covariates evaluated only explained about 40% of the observed variance. Other important factors, such as health system resilience, structure of TB services, comorbidities, population density, and poverty level are likely to be important covariates for any model that accurately predicts the variation in TB case notification we described in the face of the pandemic. As further data becomes available from other high burden TB countries on TB case notification and specific covariates, a better characterization of the association between a set of covariates and an increased gap in TB case notification can allow for better estimates of missed TB cases and inform policy that attempts to accelerate identification and treatment of these missed individuals.

## Limitations

Multiple potential limitations exist. First, more robust data on reported cases, including sociodemographic data, patient comorbidities, and residence (rural vs. urban, etc.) will improve the understanding of where these gaps in case notification are greatest and allow for targeted policies to address these findings. Second, improved data on TB care outcomes among individuals notified will allow a better characterization of how COVID-19 suppression measures have impacted patients who access treatment within the TB treatment program. Third, data are needed from areas with high rates of TB–HIV co-infection and drug resistant TB to better characterize how these gaps may differ in these groups. Fourth, we had limited specific socioeconomic and demographic data by state/territory during the pandemic, and it is likely these factors play a significant role in influencing heterogeneity in TB reporting. Fifth, our model does not address how COVID-19 suppression measures may have impacted TB transmission and future analysis will benefit from TB surveillance studies. Lastly, data is needed from more countries in a timely way to improve our understanding of how gaps in TB case notification vary by geographic region.

## Conclusion

In this study, we identified over 1.3 million fewer TB cases were reported in India than would have been expected in the absence of the COVID-19 pandemic. Decreased mobility and increased COVID-19 related hospital admissions per 100,000 population were correlated with an increased difference between reported TB cases and those expected. These variables may be surrogate measures for disruption in TB diagnostic services or, alternatively, the impact of mask use and decreased mobility on transmission dynamics. Further research is needed to clarify this association, and to identify other key contributors to the observed gap in TB case notification in India during the pandemic.

## Supplementary Information

**Additional file 1: Appendix S1.** Subnational State and Territory Case Notification and counterfactual expected cases.

**Additional file 2: Appendix S2.** Additional covariate description and model analyses.

## Data Availability

All data uses in this analysis was publicly available through the Nikshay and IHME [9, 15].

## References

[1] GBD 2019 Tuberculosis Collaborators (2021). Global, regional, and national sex differences in the global burden of tuberculosis by HIV status, 1990–2019: results from the Global Burden of Disease Study 2019. Lancet Infect Dis.

[2] World Health Organization (2020). Global tuberculosis report 2020.

[3] Worldbank. World population, total. 2021. https://data.worldbank.org/indicator/SP.POP.TOTL?most_recent_value_desc=true. Accessed 12 May 2020.

[4] World Health Organization (2019). Global tuberculosis report: executive summary 2019.

[5] Migliori GB (2020). Worldwide effects of coronavirus disease pandemic on tuberculosis services, January-April 2020. Emerg Infect Dis.

[6] Stop TB Partnership (2021). One year on, new data show global impact of COVID-19 on TB epidemic is worse than expected.

[7] Cilloni L (2020). The potential impact of the COVID-19 pandemic on the tuberculosis epidemic a modelling analysis. EClinicalMedicine.

[8] Hogan AB (2020). Potential impact of the COVID-19 pandemic on HIV, tuberculosis, and malaria in low-income and middle-income countries: a modelling study. Lancet Glob Health.

[9] National Tuberculosis Elimination Program, I.N. Nikshay report. 2020. https://reports.nikshay.in/Reports/TBNotification. Accessed 21 Mar 2021.

[10] Behera D, Sharma PP (2011). A retrospective study of seasonal variation in the number of cases diagnosed at a tertiary care tuberculosis hospital. Indian J Chest Dis Allied Sci.

[11] Kumar V (2014). Seasonality of tuberculosis in Delhi, India: a time series analysis. Tuberc Res Treat.

[12] Narula P (2015). Analyzing seasonality of tuberculosis across Indian states and union territories. J Epidemiol Glob Health.

[13] Ihme Covid- Forecasting Team (2021). Modeling COVID-19 scenarios for the United States. Nat Med.

[14] Collaborators GBDD (2020). Global age-sex-specific fertility, mortality, healthy life expectancy (HALE), and population estimates in 204 countries and territories, 1950–2019: a comprehensive demographic analysis for the Global Burden of Disease Study 2019. Lancet.

[15] IHME. COVID-19 estimates downloads. http://www.healthdata.org/covid/data-downloads. Accessed 7 May 2021.

[16] Deo S (2019). What would it cost to scale-up private sector engagement efforts for tuberculosis care? Evidence from three pilot programs in India. PLoS ONE.

[17] Wakefield J (2013). Bayesian and frequentist regression methods.

[18] Stata. VCE-options Variance estimators. https://www.stata.com/manuals13/xtvce_options.pdf. Accessed 18 Nov 2021.

[19] Davillas A, Jones AM (2020). Ex ante inequality of opportunity in health, decomposition and distributional analysis of biomarkers. J Health Econ.

[20] Hierro LA, Gomez-Alvarez R, Atienza P (2014). A consistent decomposition of the redistributive, vertical, and horizontal effects of health care finance by factor components. Health Econ.

[21] Buonsenso D (2021). COVID-19 effects on tuberculosis care in Sierra Leone. Pulmonology.

[22] Komiya K (2020). The COVID-19 pandemic and the true incidence of Tuberculosis in Japan. J Infect.

[23] Kwak N, Hwang SS, Yim JJ (2020). Effect of COVID-19 on tuberculosis notification, South Korea. Emerg Infect Dis.

[24] Lai CC, Yu WL (2020). The COVID-19 pandemic and tuberculosis in Taiwan. J Infect.

[25] Liu Q (2020). Collateral impact of the Covid-19 pandemic on tuberculosis control in Jiangsu Province, China. Clin Infect Dis.

[26] Feng L (2021). Impact of COVID-19 outbreaks and interventions on influenza in China and the United States. Nat Commun.

[27] Dharmadhikari AS (2012). Surgical face masks worn by patients with multidrug-resistant tuberculosis: impact on infectivity of air on a hospital ward. Am J Respir Crit Care Med.

[28] Fox GJ (2021). The effectiveness of individual and environmental infection control measures in reducing the transmission of *Mycobacterium tuberculosis*: a systematic review. Clin Infect Dis.

[29] Harries AD (2002). Preventing tuberculosis among health workers in Malawi. Bull World Health Organ.

[30] Moro ML (2000). Effectiveness of infection control measures in controlling a nosocomial outbreak of multidrug-resistant tuberculosis among HIV patients in Italy. Int J Tuberc Lung Dis.

[31] Roth VR (2005). A multicenter evaluation of tuberculin skin test positivity and conversion among health care workers in Brazilian hospitals. Int J Tuberc Lung Dis.

[32] Yanai H (2003). Risk of Mycobacterium tuberculosis infection and disease among health care workers, Chiang Rai, Thailand. Int J Tuberc Lung Dis.

[33] Martinez L (2017). Transmission of *Mycobacterium**Tuberculosis* in households and the community: a systematic review and meta-analysis. Am J Epidemiol.

[34] Verver S (2004). Proportion of tuberculosis transmission that takes place in households in a high-incidence area. Lancet.

[35] Nouvellet P (2021). Reduction in mobility and COVID-19 transmission. Nat Commun.

