# Supplementary material for: The impact of the COVID-19 pandemic and associated suppression measures on the burden of tuberculosis in India
Source: BMC Infect Dis. 2022 Jan 27;22:92. doi: 10.1186/s12879-022-07078-y (PMC8792515; doi:10.1186/s12879-022-07078-y)
Supplement: Supplementary file 1 — Additional file 1: Appendix S1. Subnational State and Territory Case Notification and counterfactual expected cases. [file 12879_2022_7078_MOESM1_ESM.docx]

Appendix S1

Subnational State and Territory Case Notification and counterfactual expected cases.


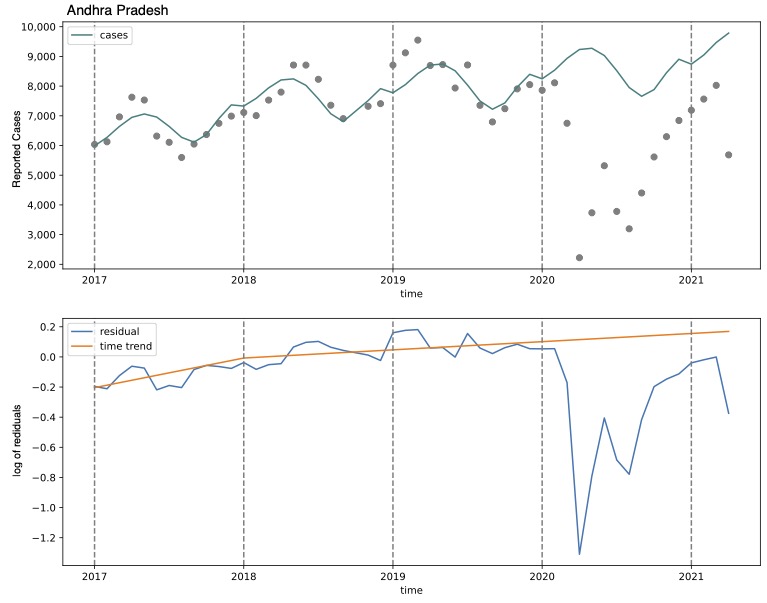


Figure A1: Case notifications and counterfactual expected cases for Andhra Pradesh

Top Panel - Case notification for Andhra Pradesh from January 2017 to April 2021. Grey points represent case notification by month. The teal line represents a model fit to case notification seasonal and year trends from January 2017 to February 2020, prior to pandemic lockdown measures in March of 2020. This trend was then extended from March 2020 to April 2021 as the counterfactual expected cases in the absence of the pandemic (March 2020 to April 2021). Bottom panel - time trend of expected cases (orange line) fit to residuals (blue line) in Andhra Pradesh, January 2017 to April 2021.


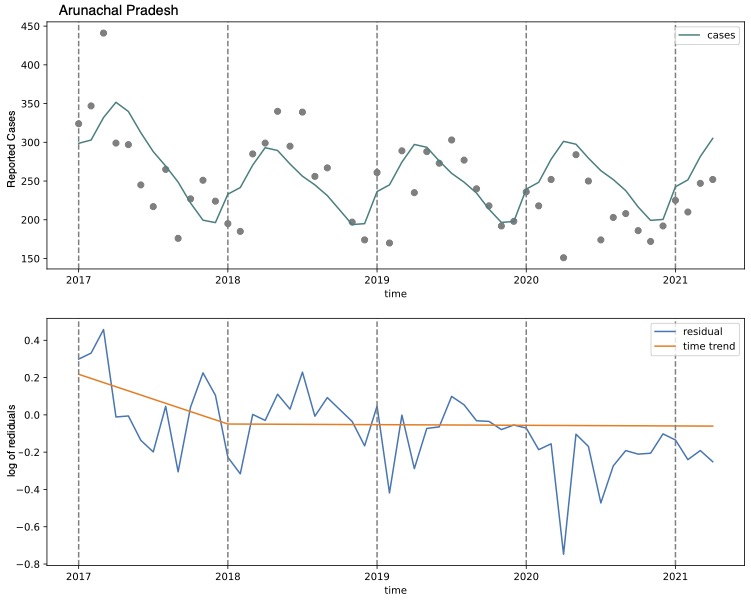


Figure A2: Case notifications and counterfactual expected cases for Arunachal Pradesh

Top Panel - Case notification for Arunachal Pradesh from January 2017 to April 2021. Grey points represent case notification by month. The teal line represents a model fit to case notification seasonal and year trends from January 2017 to February 2020, prior to pandemic lockdown measures in March of 2020. This trend was then extended from March 2020 to April 2021 as the counterfactual expected cases in the absence of the pandemic (March 2020 to April 2021). Bottom panel - time trend of expected cases (orange line) fit to residuals (blue line) in Arunachal Pradesh, January 2017 to April 2021.


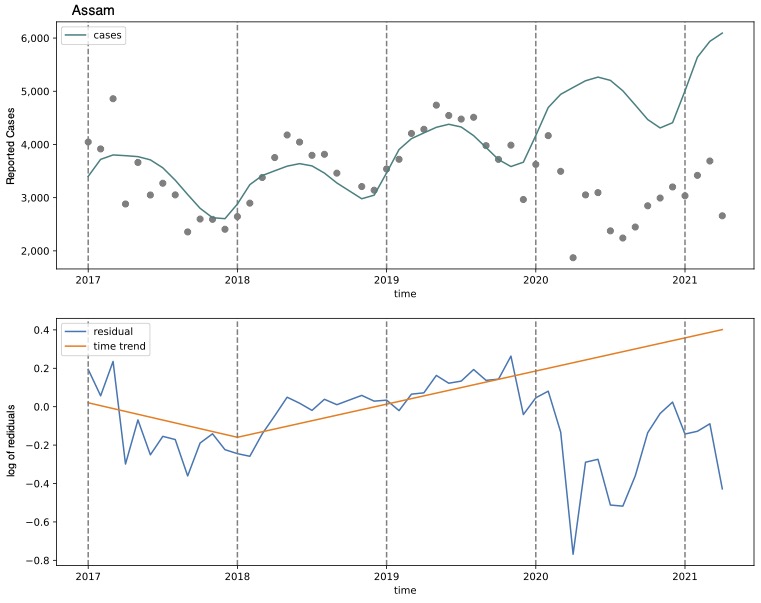


Figure A3: Case notifications and counterfactual expected cases for Assam

Top Panel - Case notification for Assam from January 2017 to April 2021. Grey points represent case notification by month. The teal line represents a model fit to case notification seasonal and year trends from January 2017 to February 2020, prior to pandemic lockdown measures in March of 2020. This trend was then extended from March 2020 to April 2021 as the counterfactual expected cases in the absence of the pandemic (March 2020 to April 2021). Bottom panel - time trend of expected cases (orange line) fit to residuals (blue line) in Assam, January 2017 to April 2021.


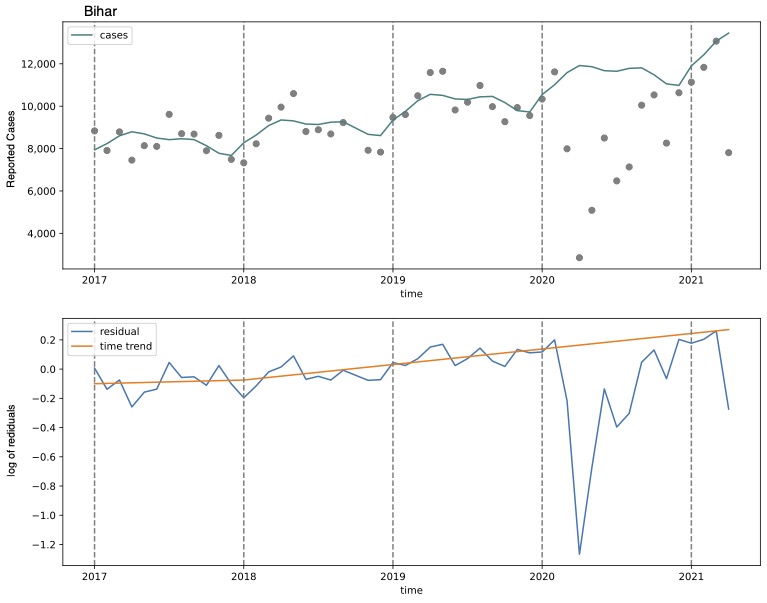


Figure A4: Case notifications and counterfactual expected cases for Bihar

Top Panel - Case notification for Bihar from January 2017 to April 2021. Grey points represent case notification by month. The teal line represents a model fit to case notification seasonal and year trends from January 2017 to February 2020, prior to pandemic lockdown measures in March of 2020. This trend was then extended from March 2020 to April 2021 as the counterfactual expected cases in the absence of the pandemic (March 2020 to April 2021). Bottom panel - time trend of expected cases (orange line) fit to residuals (blue line) in Bihar, January 2017 to April 2021.


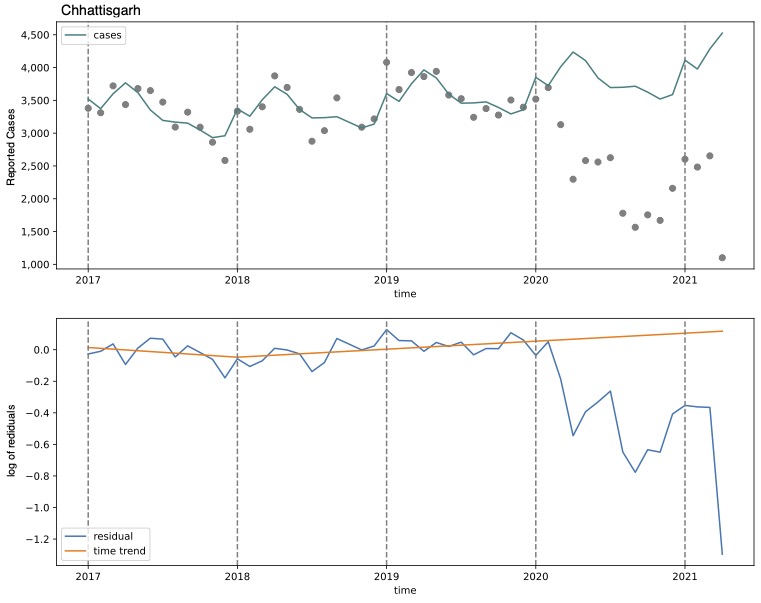


Figure A5: Case notifications and counterfactual expected cases for Chhattisgarh

Top Panel - Case notification for Chhattisgarh from January 2017 to April 2021. Grey points represent case notification by month. The teal line represents a model fit to case notification seasonal and year trends from January 2017 to February 2020, prior to pandemic lockdown measures in March of 2020. This trend was then extended from March 2020 to April 2021 as the counterfactual expected cases in the absence of the pandemic (March 2020 to April 2021). Bottom panel - time trend of expected cases (orange line) fit to residuals (blue line) in Chhattisgarh, January 2017 to April 2021.


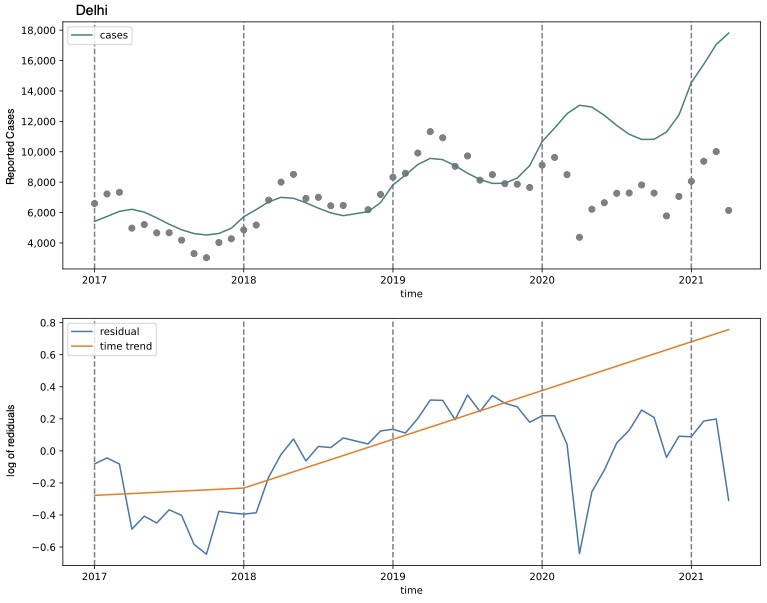


Figure A6: Case notifications and counterfactual expected cases for Delhi

Top Panel - Case notification for Delhi from January 2017 to April 2021. Grey points represent case notification by month. The teal line represents a model fit to case notification seasonal and year trends from January 2017 to February 2020, prior to pandemic lockdown measures in March of 2020. This trend was then extended from March 2020 to April 2021 as the counterfactual expected cases in the absence of the pandemic (March 2020 to April 2021). Bottom panel - time trend of expected cases (orange line) fit to residuals (blue line) in Delhi, January 2017 to April 2021.


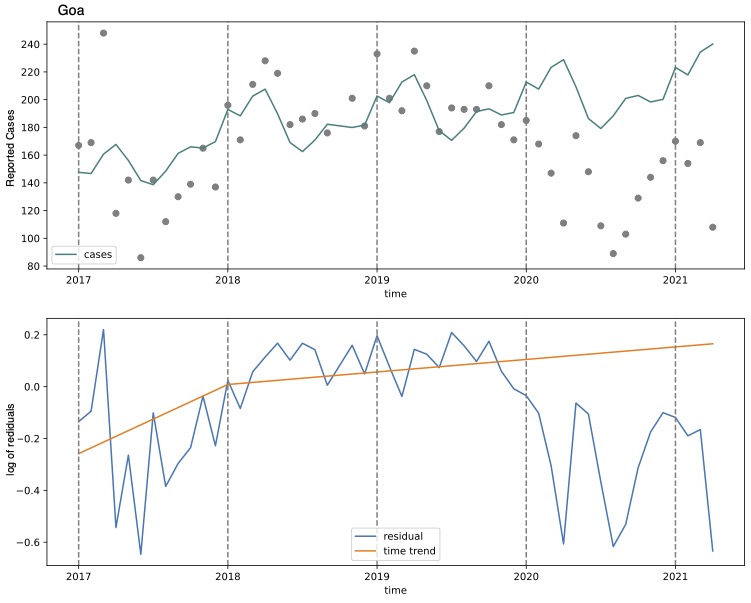


Figure A7: Case notifications and counterfactual expected cases for Goa

Top Panel - Case notification for Goa from January 2017 to April 2021. Grey points represent case notification by month. The teal line represents a model fit to case notification seasonal and year trends from January 2017 to February 2020, prior to pandemic lockdown measures in March of 2020. This trend was then extended from March 2020 to April 2021 as the counterfactual expected cases in the absence of the pandemic (March 2020 to April 2021). Bottom panel - time trend of expected cases (orange line) fit to residuals (blue line) in Goa, January 2017 to April 2021.


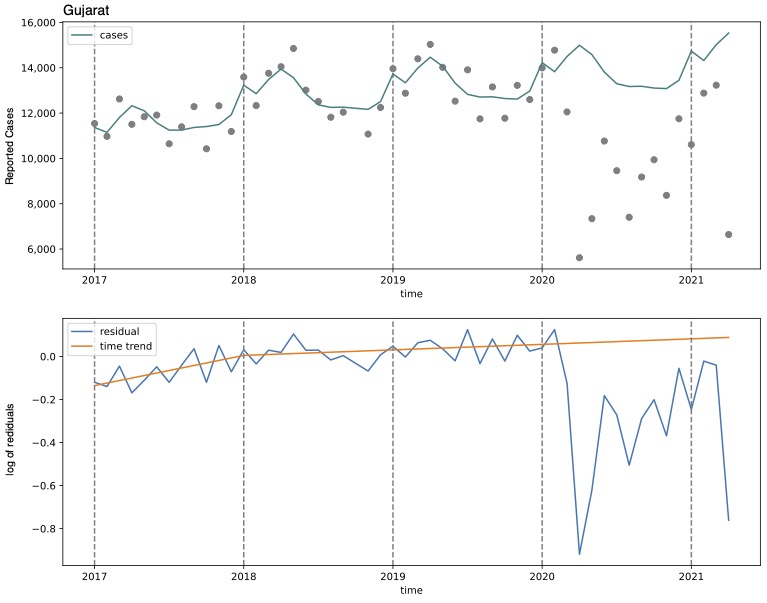


Figure A8: Case notifications and counterfactual expected cases for Gujarat

Top Panel - Case notification for Gujarat from January 2017 to April 2021. Grey points represent case notification by month. The teal line represents a model fit to case notification seasonal and year trends from January 2017 to February 2020, prior to pandemic lockdown measures in March of 2020. This trend was then extended from March 2020 to April 2021 as the counterfactual expected cases in the absence of the pandemic (March 2020 to April 2021). Bottom panel - time trend of expected cases (orange line) fit to residuals (blue line) in Gujarat, January 2017 to April 2021.


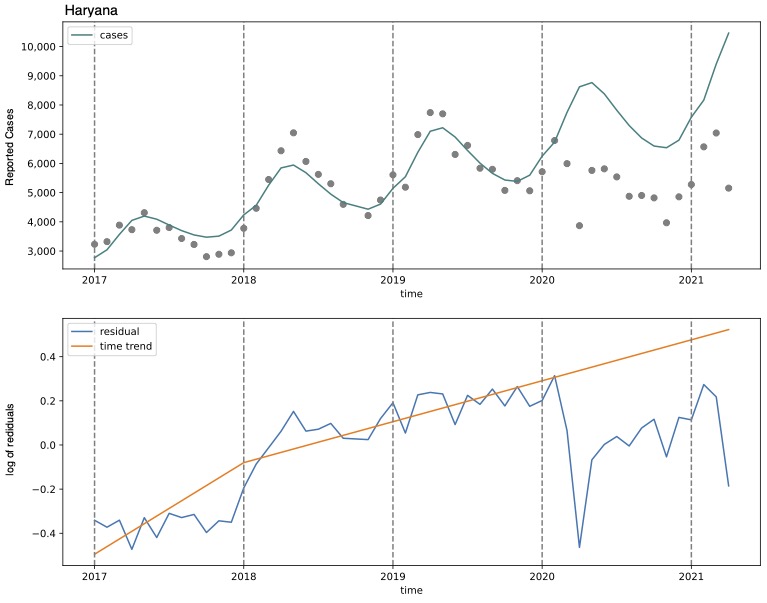


Figure A9: Case notifications and counterfactual expected cases for Haryana

Top Panel - Case notification for Haryana from January 2017 to April 2021. Grey points represent case notification by month. The teal line represents a model fit to case notification seasonal and year trends from January 2017 to February 2020, prior to pandemic lockdown measures in March of 2020. This trend was then extended from March 2020 to April 2021 as the counterfactual expected cases in the absence of the pandemic (March 2020 to April 2021). Bottom panel - time trend of expected cases (orange line) fit to residuals (blue line) in Haryana, January 2017 to April 2021.


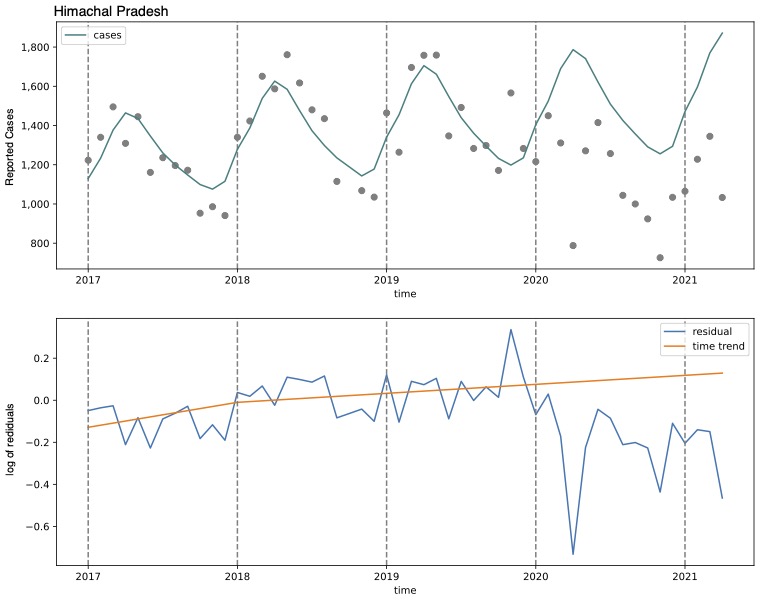


Figure A10: Case notifications and counterfactual expected cases for Himachal Pradesh

Top Panel - Case notification for Himachal Pradesh from January 2017 to April 2021. Grey points represent case notification by month. The teal line represents a model fit to case notification seasonal and year trends from January 2017 to February 2020, prior to pandemic lockdown measures in March of 2020. This trend was then extended from March 2020 to April 2021 as the counterfactual expected cases in the absence of the pandemic (March 2020 to April 2021). Bottom panel - time trend of expected cases (orange line) fit to residuals (blue line) in Himachal Pradesh, January 2017 to April 2021.


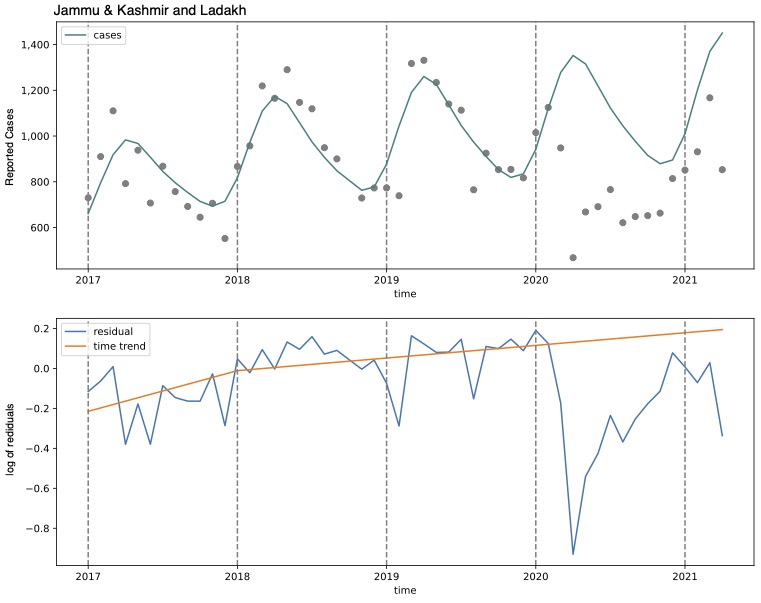


Figure A11: Case notifications and counterfactual expected cases for Jammu, Kashmir, and Ladakh

Top Panel - Case notification for Jammu, Kashmir, and Ladakh from January 2017 to April 2021. Grey points represent case notification by month. The teal line represents a model fit to case notification seasonal and year trends from January 2017 to February 2020, prior to pandemic lockdown measures in March of 2020. This trend was then extended from March 2020 to April 2021 as the counterfactual expected cases in the absence of the pandemic (March 2020 to April 2021). Bottom panel - time trend of expected cases (orange line) fit to residuals (blue line) in Jammu, Kashmir, and Ladakh, January 2017 to April 2021.


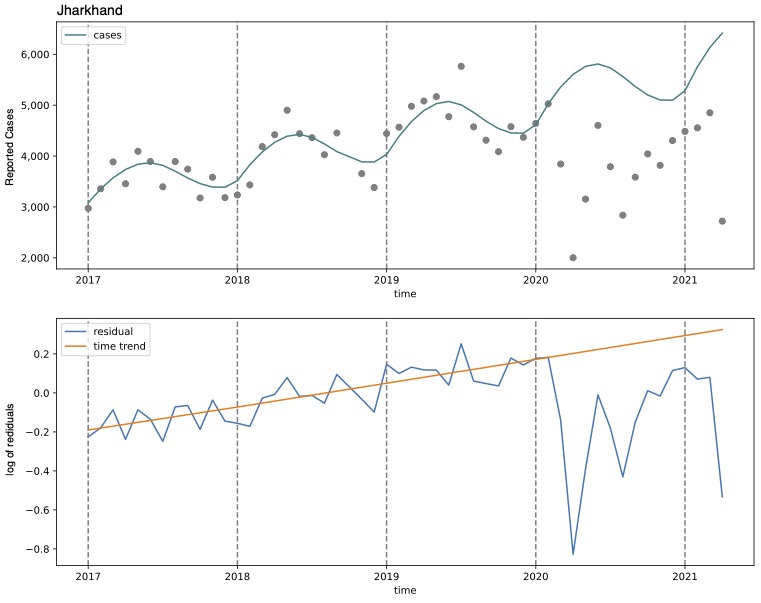


Figure A12: Case notifications and counterfactual expected cases for Jharkhand

Top Panel - Case notification for Jharkhand from January 2017 to April 2021. Grey points represent case notification by month. The teal line represents a model fit to case notification seasonal and year trends from January 2017 to February 2020, prior to pandemic lockdown measures in March of 2020. This trend was then extended from March 2020 to April 2021 as the counterfactual expected cases in the absence of the pandemic (March 2020 to April 2021). Bottom panel - time trend of expected cases (orange line) fit to residuals (blue line) in Jharkhand, January 2017 to April 2021.


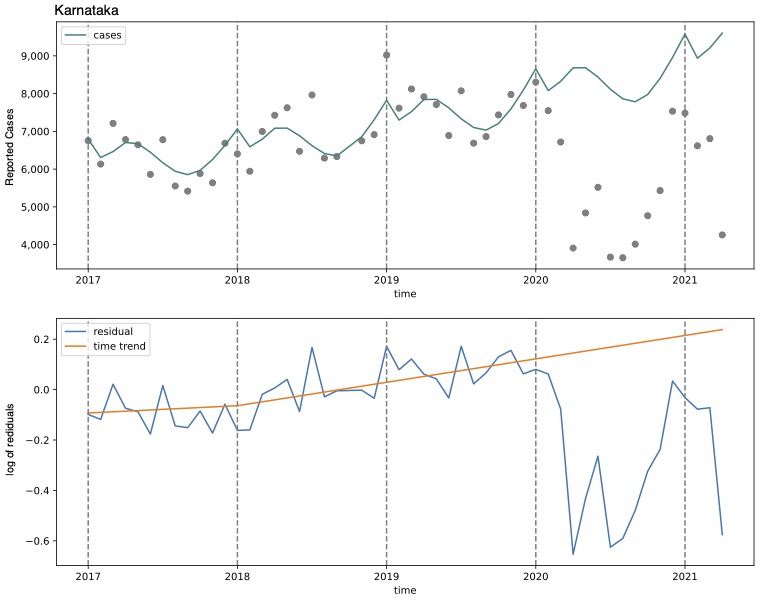


Figure A13: Case notifications and counterfactual expected cases for Karnataka

Top Panel - Case notification for Karnataka from January 2017 to April 2021. Grey points represent case notification by month. The teal line represents a model fit to case notification seasonal and year trends from January 2017 to February 2020, prior to pandemic lockdown measures in March of 2020. This trend was then extended from March 2020 to April 2021 as the counterfactual expected cases in the absence of the pandemic (March 2020 to April 2021). Bottom panel - time trend of expected cases (orange line) fit to residuals (blue line) in Karnataka, January 2017 to April 2021.


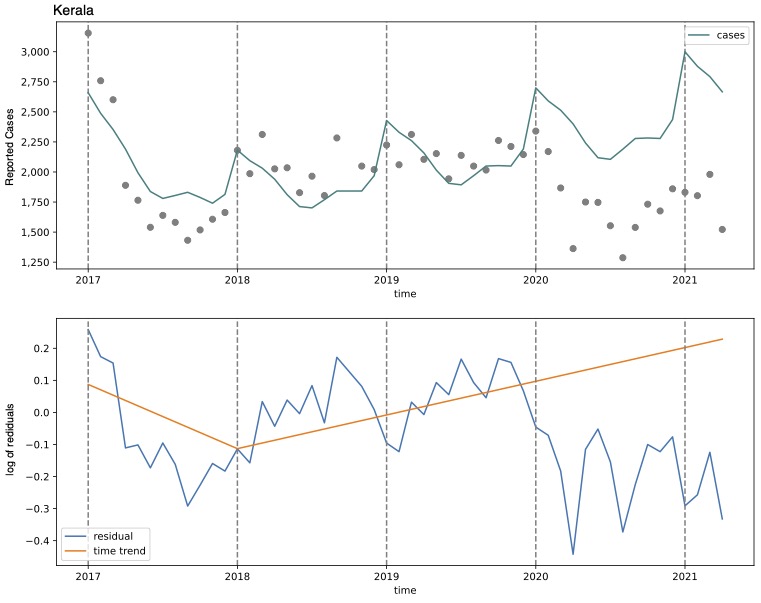


Figure A14: Case notifications and counterfactual expected cases for Kerala

Top Panel - Case notification for Kerala from January 2017 to April 2021. Grey points represent case notification by month. The teal line represents a model fit to case notification seasonal and year trends from January 2017 to February 2020, prior to pandemic lockdown measures in March of 2020. This trend was then extended from March 2020 to April 2021 as the counterfactual expected cases in the absence of the pandemic (March 2020 to April 2021). Bottom panel - time trend of expected cases (orange line) fit to residuals (blue line) in Kerala, January 2017 to April 2021.


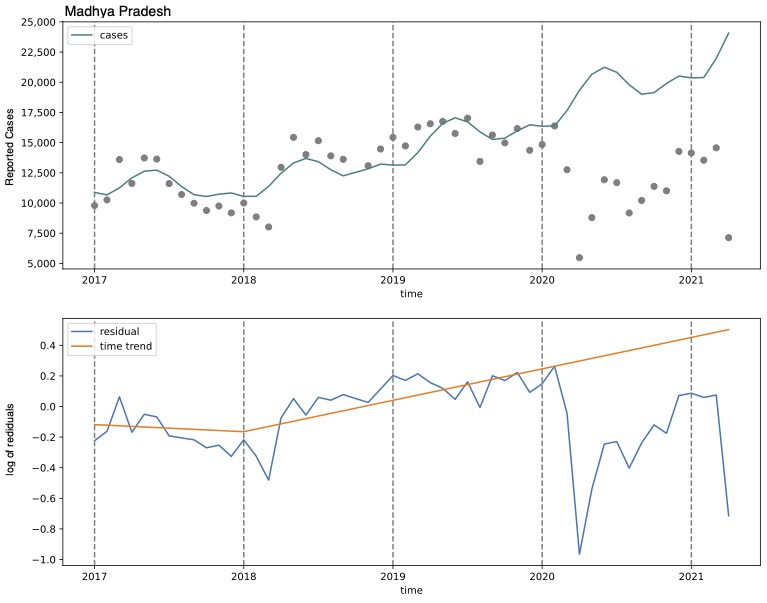


Figure A15: Case notifications and counterfactual expected cases for Madhya Pradesh

Top Panel - Case notification for Madhya Pradesh from January 2017 to April 2021. Grey points represent case notification by month. The teal line represents a model fit to case notification seasonal and year trends from January 2017 to February 2020, prior to pandemic lockdown measures in March of 2020. This trend was then extended from March 2020 to April 2021 as the counterfactual expected cases in the absence of the pandemic (March 2020 to April 2021). Bottom panel - time trend of expected cases (orange line) fit to residuals (blue line) in Madhya Pradesh, January 2017 to April 2021.


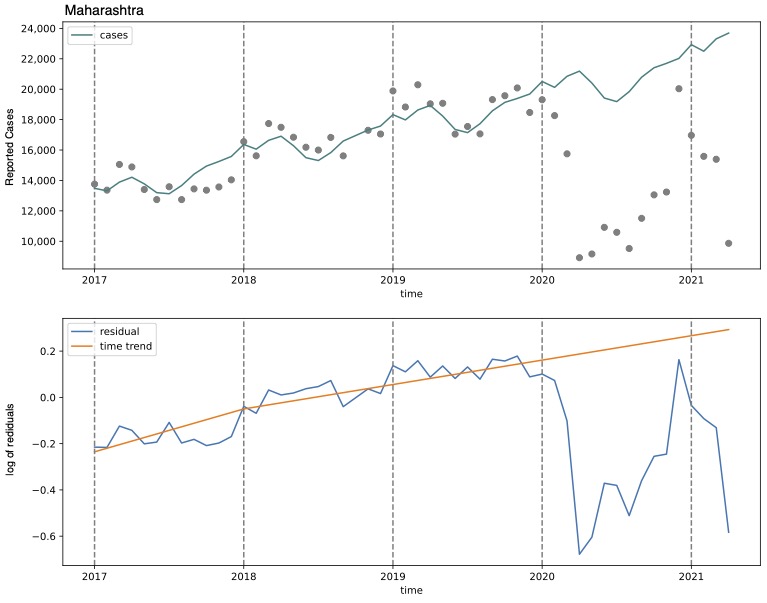


Figure A16: Case notifications and counterfactual expected cases for Maharashtra

Top Panel - Case notification for Maharashtra from January 2017 to April 2021. Grey points represent case notification by month. The teal line represents a model fit to case notification seasonal and year trends from January 2017 to February 2020, prior to pandemic lockdown measures in March of 2020. This trend was then extended from March 2020 to April 2021 as the counterfactual expected cases in the absence of the pandemic (March 2020 to April 2021). Bottom panel - time trend of expected cases (orange line) fit to residuals (blue line) in Maharashtra, January 2017 to April 2021.


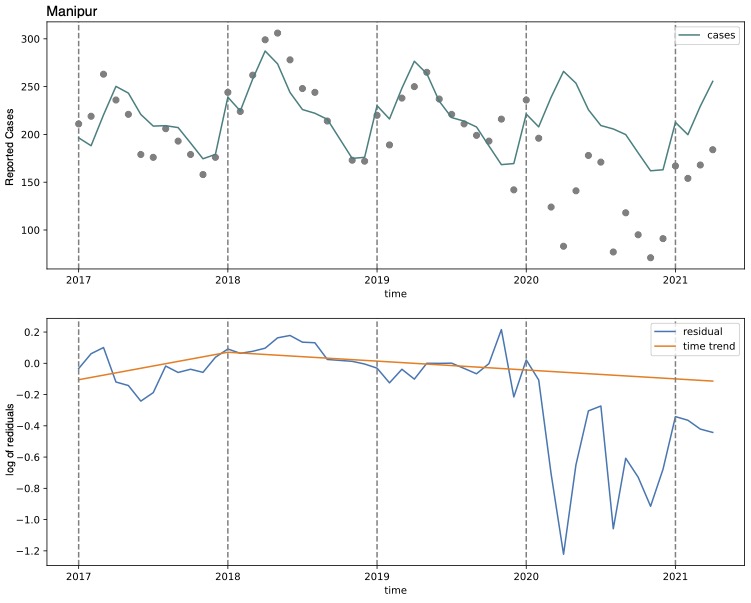


Figure A17: Case notifications and counterfactual expected cases for Manipur

Top Panel - Case notification for Manipur from January 2017 to April 2021. Grey points represent case notification by month. The teal line represents a model fit to case notification seasonal and year trends from January 2017 to February 2020, prior to pandemic lockdown measures in March of 2020. This trend was then extended from March 2020 to April 2021 as the counterfactual expected cases in the absence of the pandemic (March 2020 to April 2021). Bottom panel - time trend of expected cases (orange line) fit to residuals (blue line) in Manipur, January 2017 to April 2021.


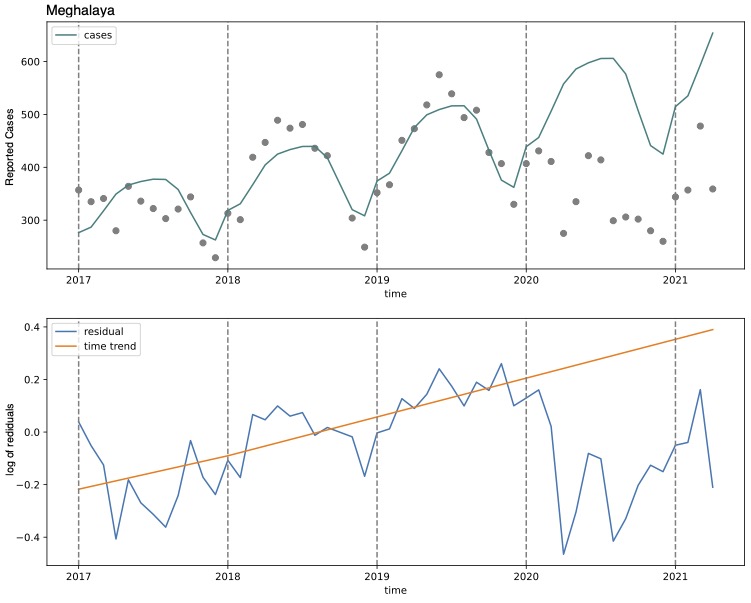


Figure A18: Case notifications and counterfactual expected cases for Meghalaya

Top Panel - Case notification for Meghalaya from January 2017 to April 2021. Grey points represent case notification by month. The teal line represents a model fit to case notification seasonal and year trends from January 2017 to February 2020, prior to pandemic lockdown measures in March of 2020. This trend was then extended from March 2020 to April 2021 as the counterfactual expected cases in the absence of the pandemic (March 2020 to April 2021). Bottom panel - time trend of expected cases (orange line) fit to residuals (blue line) in Meghalaya, January 2017 to April 2021.


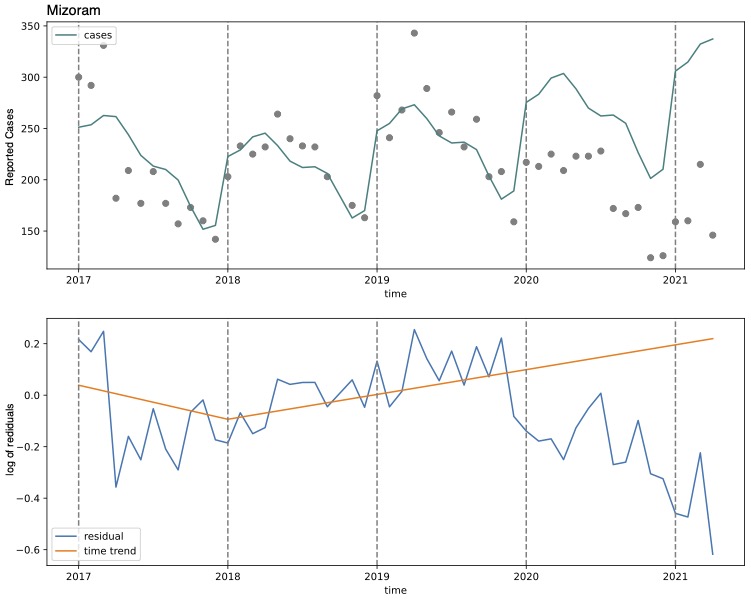


Figure A19: Case notifications and counterfactual expected cases for Mizoram

Top Panel - Case notification for Mizoram from January 2017 to April 2021. Grey points represent case notification by month. The teal line represents a model fit to case notification seasonal and year trends from January 2017 to February 2020, prior to pandemic lockdown measures in March of 2020. This trend was then extended from March 2020 to April 2021 as the counterfactual expected cases in the absence of the pandemic (March 2020 to April 2021). Bottom panel - time trend of expected cases (orange line) fit to residuals (blue line) in Mizoram, January 2017 to April 2021.


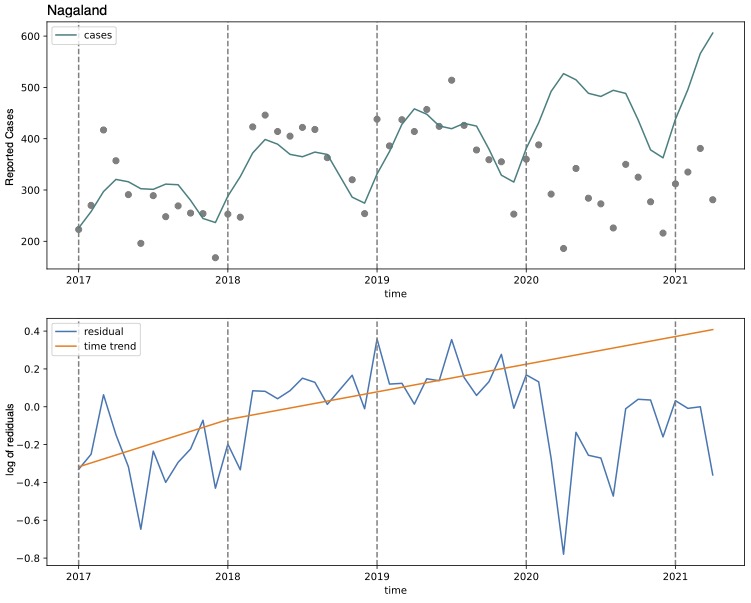


Figure A20: Case notifications and counterfactual expected cases for Nagaland

Top Panel - Case notification for Nagaland from January 2017 to April 2021. Grey points represent case notification by month. The teal line represents a model fit to case notification seasonal and year trends from January 2017 to February 2020, prior to pandemic lockdown measures in March of 2020. This trend was then extended from March 2020 to April 2021 as the counterfactual expected cases in the absence of the pandemic (March 2020 to April 2021). Bottom panel - time trend of expected cases (orange line) fit to residuals (blue line) in Nagaland, January 2017 to April 2021.


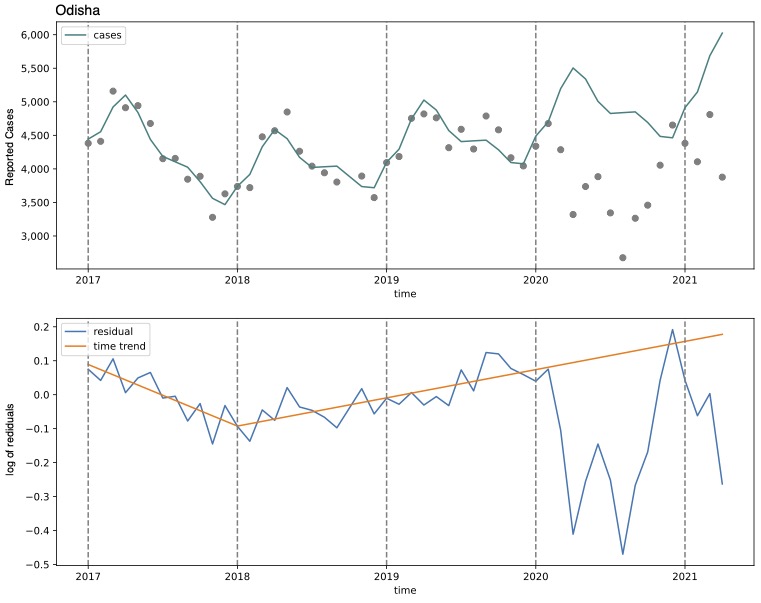


Figure A21: Case notifications and counterfactual expected cases for Odisha

Top Panel - Case notification for Odisha from January 2017 to April 2021. Grey points represent case notification by month. The teal line represents a model fit to case notification seasonal and year trends from January 2017 to February 2020, prior to pandemic lockdown measures in March of 2020. This trend was then extended from March 2020 to April 2021 as the counterfactual expected cases in the absence of the pandemic (March 2020 to April 2021). Bottom panel - time trend of expected cases (orange line) fit to residuals (blue line) in Odisha, January 2017 to April 2021.


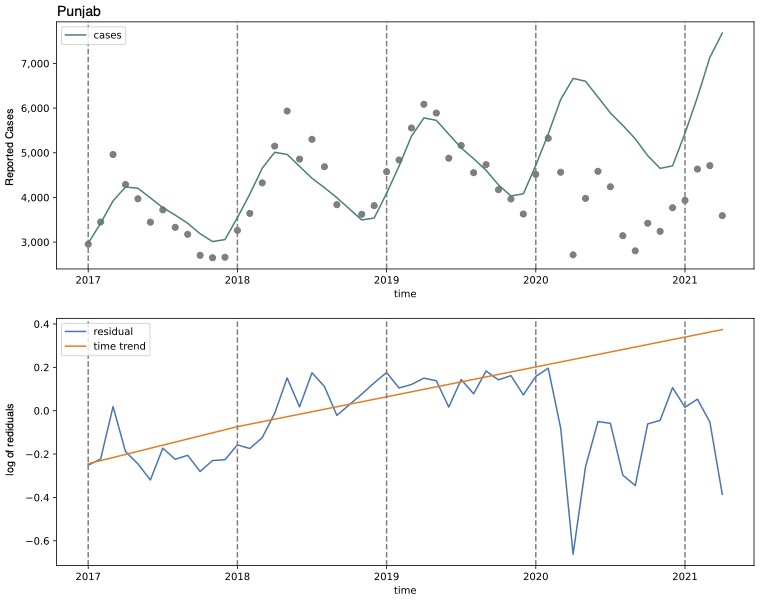


Figure A22: Case notifications and counterfactual expected cases for Punjab

Top Panel - Case notification for Punjab from January 2017 to April 2021. Grey points represent case notification by month. The teal line represents a model fit to case notification seasonal and year trends from January 2017 to February 2020, prior to pandemic lockdown measures in March of 2020. This trend was then extended from March 2020 to April 2021 as the counterfactual expected cases in the absence of the pandemic (March 2020 to April 2021). Bottom panel - time trend of expected cases (orange line) fit to residuals (blue line) in Punjab, January 2017 to April 2021.


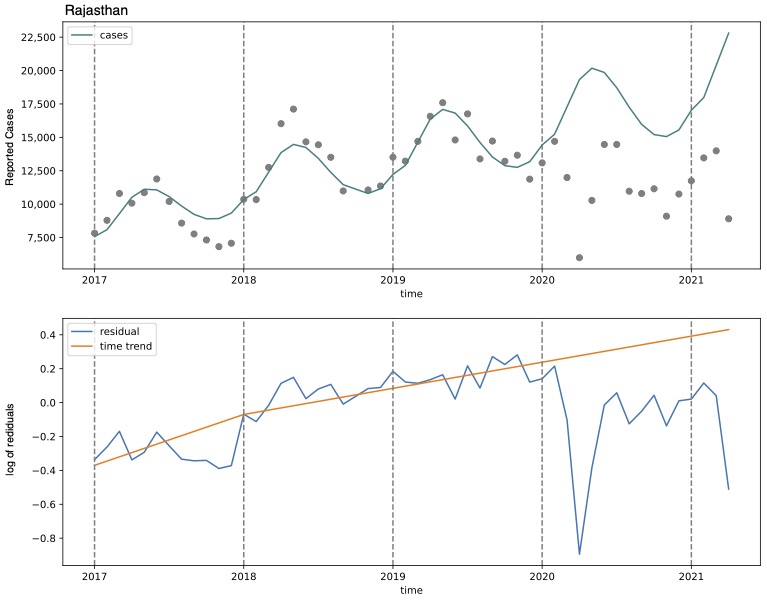


Figure A23: Case notifications and counterfactual expected cases for Rajasthan

Top Panel - Case notification for Rajasthan from January 2017 to April 2021. Grey points represent case notification by month. The teal line represents a model fit to case notification seasonal and year trends from January 2017 to February 2020, prior to pandemic lockdown measures in March of 2020. This trend was then extended from March 2020 to April 2021 as the counterfactual expected cases in the absence of the pandemic (March 2020 to April 2021). Bottom panel - time trend of expected cases (orange line) fit to residuals (blue line) in Rajasthan, January 2017 to April 2021.


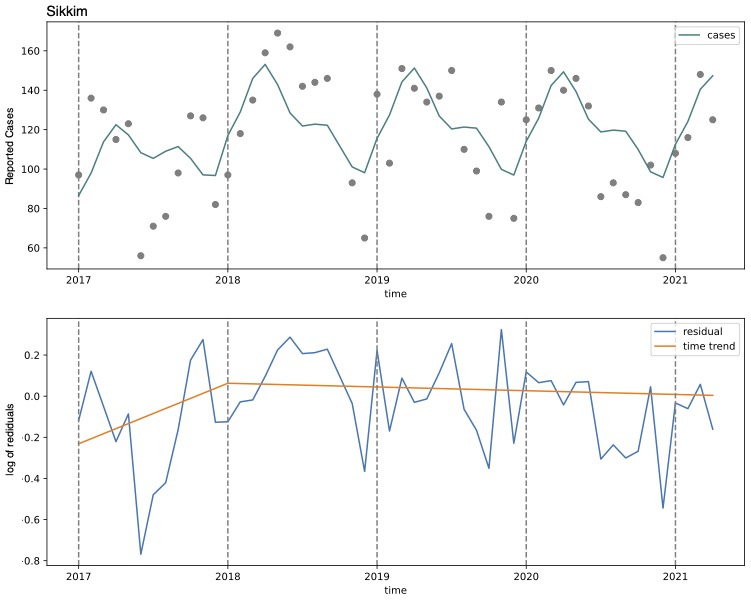


Figure A24: Case notifications and counterfactual expected cases for Sikkim

Top Panel - Case notification for Sikkim from January 2017 to April 2021. Grey points represent case notification by month. The teal line represents a model fit to case notification seasonal and year trends from January 2017 to February 2020, prior to pandemic lockdown measures in March of 2020. This trend was then extended from March 2020 to April 2021 as the counterfactual expected cases in the absence of the pandemic (March 2020 to April 2021). Bottom panel - time trend of expected cases (orange line) fit to residuals (blue line) in Sikkim, January 2017 to April 2021.


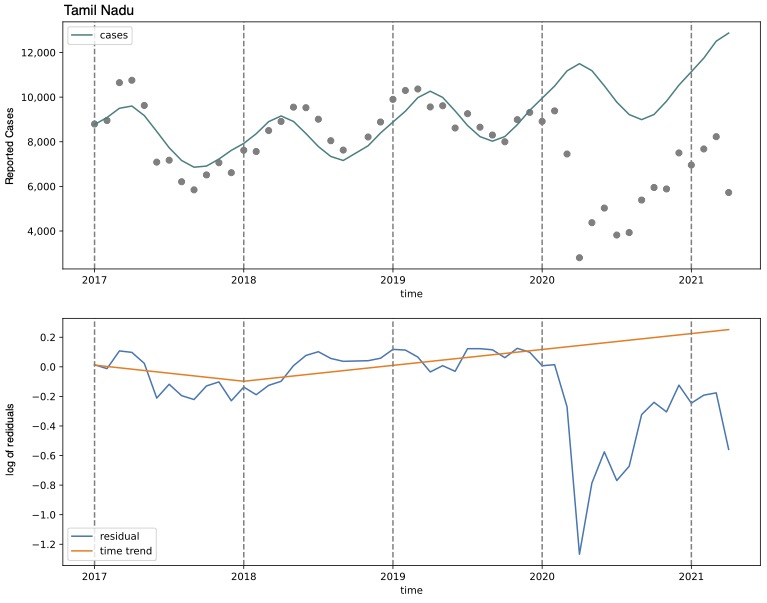


Figure A25: Case notifications and counterfactual expected cases for Tamil Nadu

Top Panel - Case notification for Tamil Nadu from January 2017 to April 2021. Grey points represent case notification by month. The teal line represents a model fit to case notification seasonal and year trends from January 2017 to February 2020, prior to pandemic lockdown measures in March of 2020. This trend was then extended from March 2020 to April 2021 as the counterfactual expected cases in the absence of the pandemic (March 2020 to April 2021). Bottom panel - time trend of expected cases (orange line) fit to residuals (blue line) in Tamil Nadu, January 2017 to April 2021.


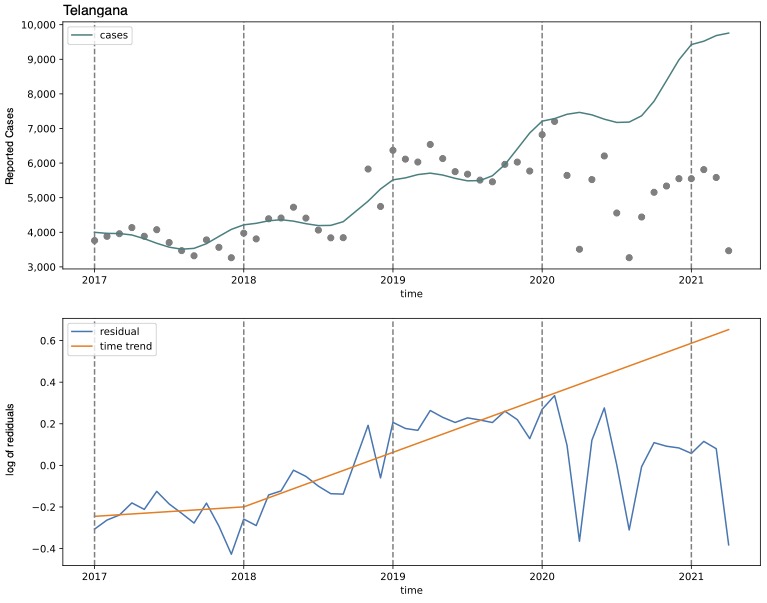


Figure A26: Case notifications and counterfactual expected cases for Telangana

Top Panel - Case notification for Telangana from January 2017 to April 2021. Grey points represent case notification by month. The teal line represents a model fit to case notification seasonal and year trends from January 2017 to February 2020, prior to pandemic lockdown measures in March of 2020. This trend was then extended from March 2020 to April 2021 as the counterfactual expected cases in the absence of the pandemic (March 2020 to April 2021). Bottom panel - time trend of expected cases (orange line) fit to residuals (blue line) in Telangana, January 2017 to April 2021.


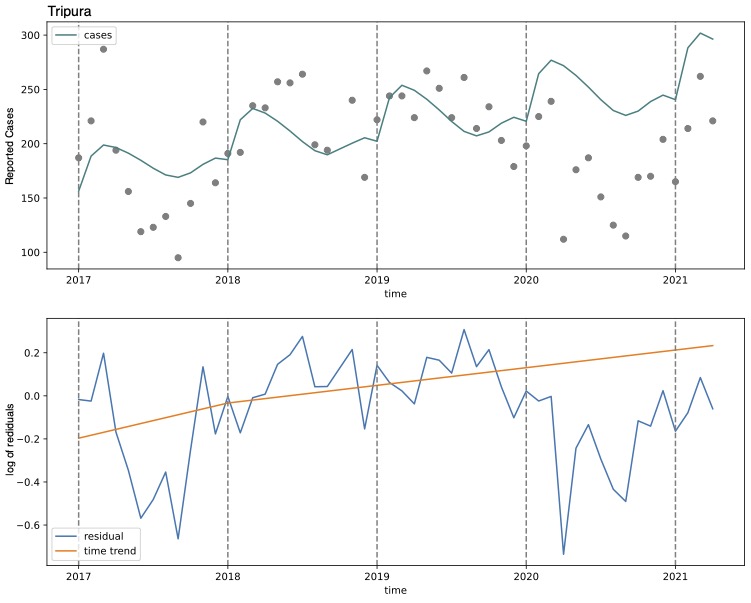


Figure A27: Case notifications and counterfactual expected cases for Tripura

Top Panel - Case notification for Tripura from January 2017 to April 2021. Grey points represent case notification by month. The teal line represents a model fit to case notification seasonal and year trends from January 2017 to February 2020, prior to pandemic lockdown measures in March of 2020. This trend was then extended from March 2020 to April 2021 as the counterfactual expected cases in the absence of the pandemic (March 2020 to April 2021). Bottom panel - time trend of expected cases (orange line) fit to residuals (blue line) in Tripura, January 2017 to April 2021.


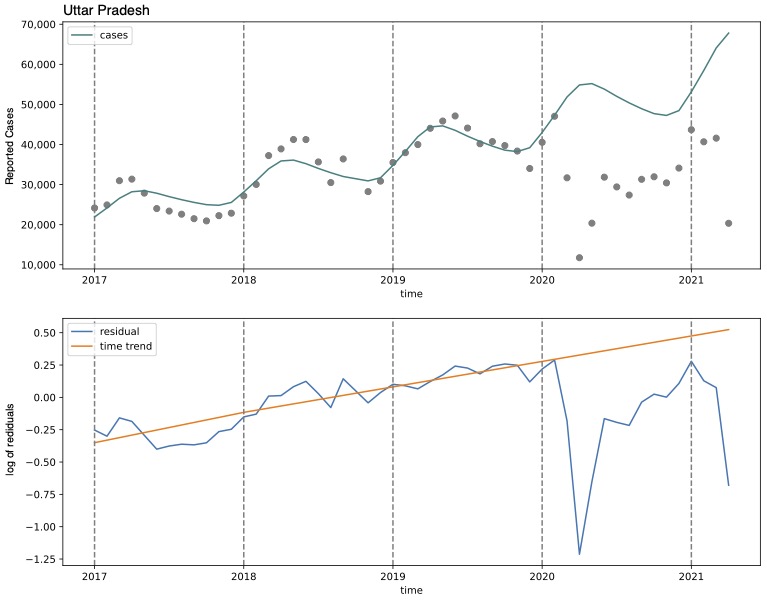


Figure A28: Case notifications and counterfactual expected cases for Uttar Pradesh

Top Panel - Case notification for Uttar Pradesh from January 2017 to April 2021. Grey points represent case notification by month. The teal line represents a model fit to case notification seasonal and year trends from January 2017 to February 2020, prior to pandemic lockdown measures in March of 2020. This trend was then extended from March 2020 to April 2021 as the counterfactual expected cases in the absence of the pandemic (March 2020 to April 2021). Bottom panel - time trend of expected cases (orange line) fit to residuals (blue line) in Uttar Pradesh, January 2017 to April 2021.


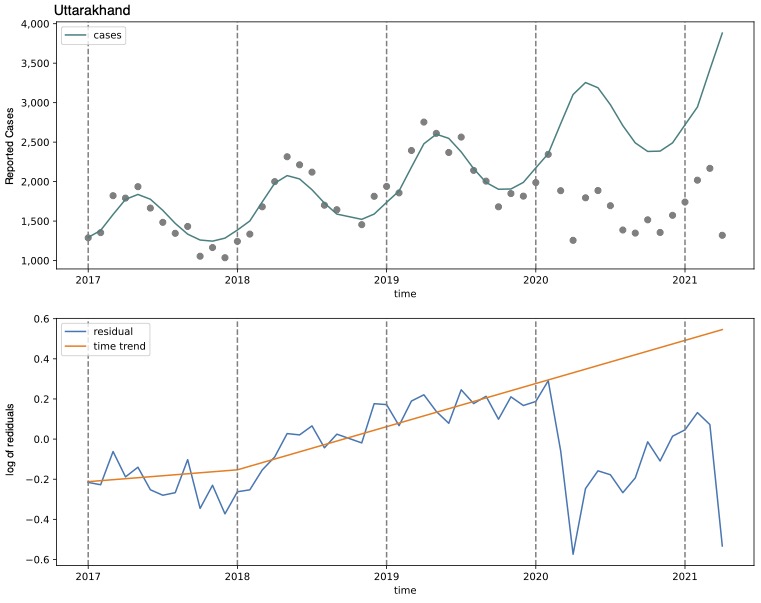


Figure A29: Case notifications and counterfactual expected cases for Uttarakhand

Top Panel - Case notification for Uttarakhand from January 2017 to April 2021. Grey points represent case notification by month. The teal line represents a model fit to case notification seasonal and year trends from January 2017 to February 2020, prior to pandemic lockdown measures in March of 2020. This trend was then extended from March 2020 to April 2021 as the counterfactual expected cases in the absence of the pandemic (March 2020 to April 2021). Bottom panel - time trend of expected cases (orange line) fit to residuals (blue line) in Uttarakhand, January 2017 to April 2021.


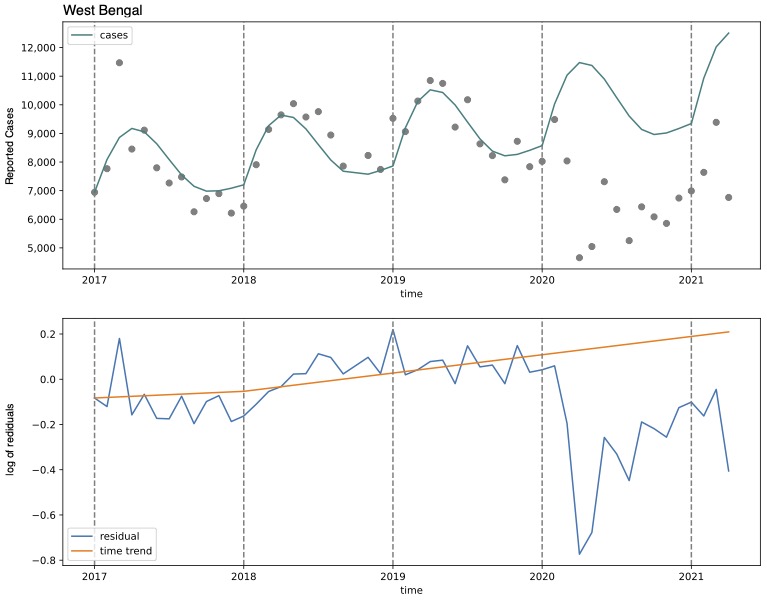


Figure A30: Case notifications and counterfactual expected cases for West Bengal

Top Panel - Case notification for West Bengal from January 2017 to April 2021. Grey points represent case notification by month. The teal line represents a model fit to case notification seasonal and year trends from January 2017 to February 2020, prior to pandemic lockdown measures in March of 2020. This trend was then extended from March 2020 to April 2021 as the counterfactual expected cases in the absence of the pandemic (March 2020 to April 2021). Bottom panel - time trend of expected cases (orange line) fit to residuals (blue line) in West Bengal, January 2017 to April 2021.


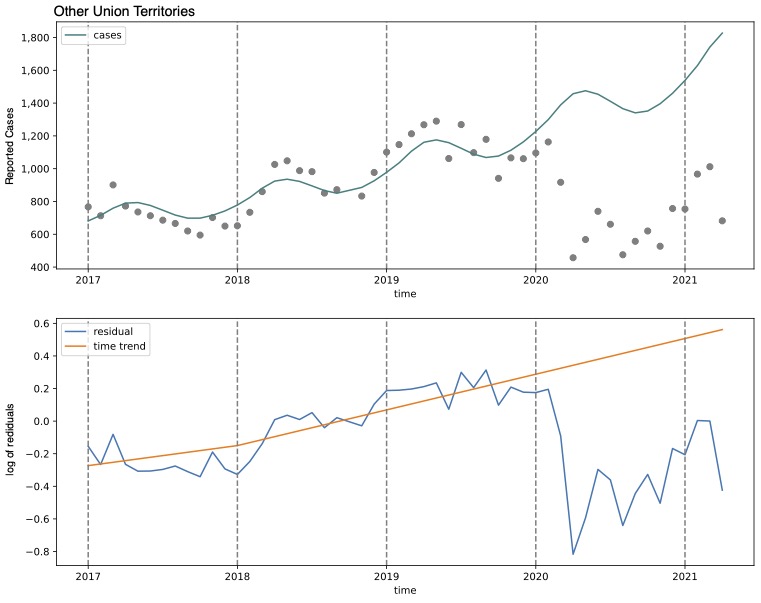


Figure A31: Case notifications and counterfactual expected cases for Union Territories not characterized in Figures A1-A30

Top Panel - Case notification for Union Territories not characterized in Figures A1-A30 from January 2017 to April 2021. Grey points represent case notification by month. The teal line represents a model fit to case notification seasonal and year trends from January 2017 to February 2020, prior to pandemic lockdown measures in March of 2020. This trend was then extended from March 2020 to April 2021 as the counterfactual expected cases in the absence of the pandemic (March 2020 to April 2021). Bottom panel - time trend of expected cases (orange line) fit to residuals (blue line) in Union Territories not characterized in Figures A1-A30, January 2017 to April 2021.
