# Supplementary material for: The impact of the COVID-19 pandemic and associated suppression measures on the burden of tuberculosis in India
Source: BMC Infect Dis. 2022 Jan 27;22:92. doi: 10.1186/s12879-022-07078-y (PMC8792515; doi:10.1186/s12879-022-07078-y)
Supplement: Supplementary file 2 — Additional file 2: Appendix S2. Additional covariate description and model analyses. [file 12879_2022_7078_MOESM2_ESM.docx]

**Appendix S2:**

**Table A1: Regression model with the stagnant assumption: March 2020 to February 2021**

| **Covariate** | **Mean** | **Min, Max** | **SD** | **Coefficient** | **P value** | **Shapley % R2** |
| --- | --- | --- | --- | --- | --- | --- |
| Mask use | 0.586 | 0.000, 0.831 | 0.210 | 0.384 | 0.272 | 0.78% |
| Mobility | -34.997 | -87.136, -5.21 | 16.279 | 0.034 | <0.001 | 87.92% |
| Hospital admissions, per 100K population | 1.636 | 0.004, 12.77 | 2.201 | -0.092 | 0.005 | 11.05% |
| Public/total case notification ratio | 0.763 | 0.420, 1.000 | 0.121 | -0.086 | 0.916 | 0.25% |

Table A1: Evaluation of covariates in the regression model using the stagnant assumptions (i.e. no change in expected TB cases from March 2019 to February 2020 and from March 2020 to February 2021). SD = standard deviation. Overall R^2^ was 32.3%. Coefficient for the regression, P value, and percentage contribution to the R^2^ for mask use, mobility, hospital admissions, and the ratio of public case notification/total case notification are demonstrated in the table. Regression was not performed on the Union Territories due to lack of available data on mobility, mask use, and COVID-19 related hospitalizations from that area.

**Table A2: Historical analysis: March 2020 to December 2020 to validate robustness of results**

| **Covariate** | **Mean** | **Min, Max** | **SD** | **Coefficient** | **P value** | **Shapley % R2** |
| --- | --- | --- | --- | --- | --- | --- |
| Mask use | 0.579 | 0.000, 0.831 | 0.226 | 0.189 | 0.401 | 2.03% |
| Mobility | -38.044 | -87.136, -5.21 | 15.967 | 0.026 | <0.001 | 84.09% |
| Hospital admissions, per 100K population | 1.88 | 0.004, 12.77 | 2.323 | -0.067 | 0.002 | 9.81% |
| Public/total case notification ratio | 0.763 | 0.420, 1.000 | 0.121 | 0.705 | 0.314 | 4.06% |

Table A2: Historical evaluation of covariates in the regression model to validate robustness of results (i.e. only through December 2020). SD = standard deviation. Overall R^2^ was 38.1%. Coefficient for the regression, P value, and percentage contribution to the R^2^ for mask use, mobility, hospital admissions, and the ratio of public case notification/total case notification are demonstrated in the table. Regression was not performed on the Union Territories due to lack of available data on mobility, mask use, and COVID-19 related hospitalizations from that area.

**Description of data processing for Mask Use and Mobility covariates^1^**

The mask use covariate represents the proportion of adults that self-report always wearing a mask when outside their homes. To evaluate mask usage in countries including India, COVID-19 symptom survey data collected via the Facebook app by the University of Maryland were used. Although the main purpose of the survey was to collect data on COVID-19-related symptoms, the responses to the following question were used for creating the mask use covariate: “In the last 7 days, how often did you wear a mask when in public?” Response options include: “All of the time; Most of the time; About half of the time; Sometimes; Never; I have not been in public during the last 7 days”.

Mobility is measured as a relative change from the pre-pandemic baseline level. Google data represent a percentage difference in visiting certain places/destinations relative to the baseline, which is the median value, for the corresponding day of the week during the period from January 3 to February 6, 2020. Data reports are stratified according to six destinations: “Retail & recreation”, “Grocery and pharmacy”, “Parks”, “Transit stations”, “Workplaces”, and “Residential”. The average of the percentage change in the “Retail & recreation”, “Transit stations”, and “Workplaces” was computed given that these three destinations represent activities most strongly impacted by the social distancing measures. Facebook measures baseline mobility as the average over the 45 days before the beginning of data collection. To enhance comparability of different baseline periods, Facebook data were adjusted to be consistent with the baseline from Google.

**Reference**

1. IHME COVID-19 Forecasting Team, Modeling COVID-19 scenarios for the United States. Nat Med, 2021. 27(1): p. 94-105.
